# An evaluation of trans-anal rectoscopic-assisted minimally invasive surgery (ARAMIS): a new platform for transanal surgery

**DOI:** 10.1007/s00384-020-03641-8

**Published:** 2020-05-24

**Authors:** Lino Polese, Roberto Rizzato, Andrea Porzionato, Gianfranco Da Dalt, Alice Bressan, Raffaele De Caro, Stefano Merigliano

**Affiliations:** 1 Department of Surgery, Oncology and Gastroenterology, Section of Surgery, University of Padova, Clinica Chirurgica 3^, Sesto Piano Policlinico, Via Giustiniani 2, 35128 Padova, Italy; 2 Department of Neuroscience, Section of Human Anatomy, University of Padova, Padova, Italy

**Keywords:** TEM, TAMIS, Rectal tumors

## Abstract

**Purpose:**

The study aimed to evaluate the feasibility and safety of a new trans-anal rectoscopic-assisted minimally invasive surgery (ARAMIS) platform to treat rectal lesions.

**Methods:**

ARAMIS was first compared with two transanal minimally invasive surgery platforms (SILS Port and GelPOINT Path) on human cadavers. Surgeons with different experience performed running sutures at different distances, at four quadrants, using the three platforms and gave a score to visibility, safety, and maneuverability. ARAMIS was then utilized on patients affected with rectal neoplasia who met the inclusion criteria. Patients and tumor characteristic and results were prospectively collected. The follow-up examinations included proctoscopy at 3, 6, and 12 months.

**Results:**

According to surgeons’ scores, ARAMIS improves visibility and safety with respect to other platforms for distances beyond 10 cm. The procedure, which lasted an average of 59 min, was successfully carried out in 14 patients. No intraoperative or postoperative complications were reported. The mean tumor size was 3 cm; they were located a mean of 11 cm from the anal verge. Complete removal of the lesion was possible in 13/14 patients. There was one case of adenoma recurrence at follow-up.

**Conclusion:**

Study results showed that ARAMIS, which is equipped with an adjustable rectoscope, can be considered a safe, effective platform for transanal surgery. The rectoscope protects the rectum during the procedure, a particularly important consideration when proximal rectal lesions are being treated. Further clinical studies are warranted to confirm these encouraging results.

## Introduction

Transanal surgery for rectal cancer is feasible and widely accepted in early stages. According to National Comprehensive Cancer Network guidelines, transanal excision (TAE) can be performed if the lesion is smaller than 3 cm and there is less than 30% of bowel circumference involvement. In addition, the rectal tumor must be stage T1, mobile and non-fixed to the rectal wall, well to moderately differentiated, located within 8 cm of the anal verge, and showing no evidence of lymphadenopathy on pretreatment imaging. A proper local excision should, according to guidelines and standard protocols, be full thickness with clear margins (more than 3 mm) [1].

The TAE technique, which is indicated in cases of early-stage rectal cancer, is able to preserve the function of the anal sphincter and is associated with early recovery and low morbidity with respect to radical rectal resection. Its limitations are that it can be used exclusively for tumors smaller than 4 cm located within 8 cm of the anal verge. From a technical viewpoint, the technique provides poor visualization, is associated to a high rate of specimen fragmentation, and negative margins have proved to be a challenge. Some studies have, in fact, reported high recurrence rates [2]. With the intent to overcome these limits, minimally invasive transanal excision of early stage rectal neoplasm can be performed also by transanal endoscopic microsurgery (TEM) and transanal minimally invasive surgery (TAMIS).

TEM was introduced by Buess et al. in 1983 [3] as an alternative technique to transanal excision. The instrument consists in a rigid proctoscope, which is 4-cm diameter in diameter and varies from 12 to 20 cm in length. It utilizes a closed proctoscopic system and a laparoscopic camera that provides high-definition magnified visualization, permitting precise excision of the middle and upper rectal wall lesions [4, 5]. TAMIS, which is a crossover between single-incision laparoscopic surgery and transanal endoscopic microsurgery, was first described in 2010 by Atallah [6]. The technique is performed using a disposable single-port device which is introduced into the anal canal using a steady manual pressure. Once the port is positioned, a pneumorectum is established, and the operation is performed with ordinary laparoscopic instruments. Currently in the USA, there are two FDA approved devices for transanal access for the TAMIS procedure: the GelPOINT Path (Applied Medical, Rancho Santa Margarita, CA) and the SILS™ Port (Covidien, Mansfield, MA). Both are easily positioned transanally and allow insufflation through a separate channel. Other devices have been proposed for TAMIS operation: Triport Access System [7], Single Site Laparoscopic Access System [8], GelPOINT Path Long Channel transanal access platform (Applied Medical, Rancho Santa Margarita, CA) [9], and Glove port [10].

TAMIS is considered an optimal procedure for the local excision of small rectal lesions, located in mean 7.6 cm from the anal verge given its rapid installation and shorter operative times with respect to TEM [11]. TAMIS and TEM appear to have same indications and outcomes although the latter provides only limited visualization and is difficult to manipulate because of a rigid side-viewing proctoscope. TAMIS, on the other hand, provides a wide, circumferential visualization of the rectum and permits greater flexibility in positioning patients [12]. No differences in the quality of the excisions or in postoperative complications were noted by one study examining large groups of patients treated with TEM or TAMIS. Although the former initially appeared to be the more expensive procedure, it is important to remember that given the high cost of TEM equipment and the steep learning curve, a cost analysis must take into consideration the volume of procedures carried out in a specific center [13].

One of TAMIS’ greatest limitation with respect to TEM is that it is not equipped with a rectoscope which normally serves to protect the rectum. To overcome this limit, we designed a disposable platform for transanal minimally invasive surgery equipped with an adjustable rectoscope. The instrument, which is called ARAMIS (trans-anal rectoscopic-assisted minimally invasive surgery), can safely host laparoscopic instrumentation such as that of the TAMIS technique. The current study sets out to evaluate feasibility and outcomes of ARAMIS by an experimental and clinical study.

## Materials and methods

### ARAMIS

**Fig. 1:**
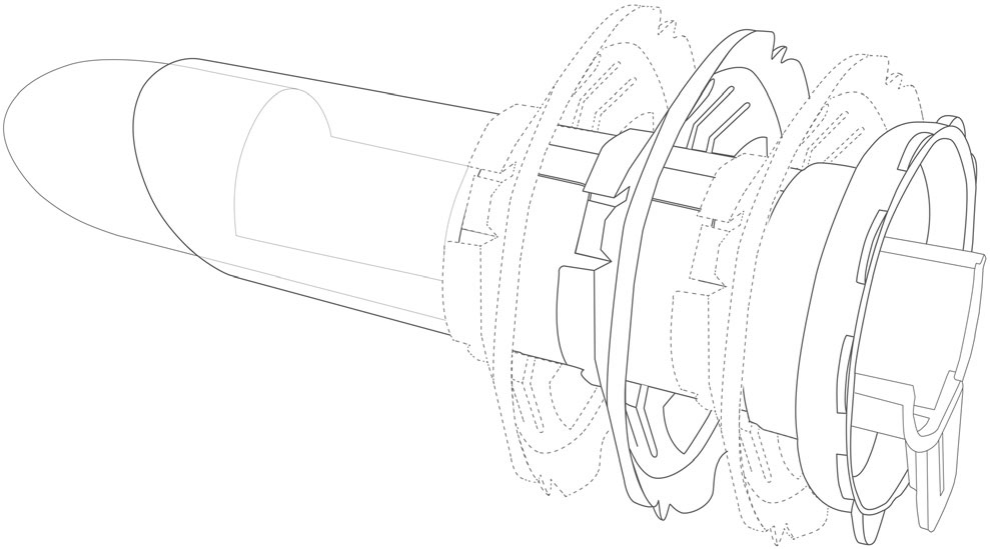
The three measures of insertion of the rectoscope

ARAMIS (SapiMed, Alessandria, Italy) is a new disposable platform for trans-anal rectoscopic-assisted minimally invasive surgery consisting of an all-in-one solution combining a single port access and a rigid rectoscope. The 110 × 35.5 mm rectoscope with a distal 45° flute opening is self-sustaining by means of a plastic fixing ring that is sutured to the perianal skin. The rectoscope is adjustable, depending on the location of the lesion, to three possibilities: 5–7 cm, 7–9 cm, and 9–11 cm from the anal verge (Fig. 1). The distal flute opening can be rotated depending on the tumor position permitting a clearer vision of the lesion with the wider opening pointed in its direction. There is a mark on the rectoscope indicating the direction of the flute opening even when the instrument is inserted transanally.

**Fig. 2:**
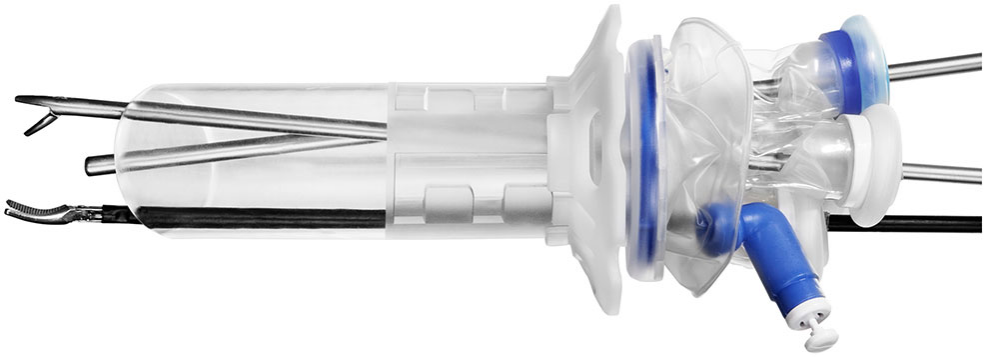
The rectoscope connected to the flexible single access surgery port (Gloveport, Nelis, Bucheon City, Korea) with four working channels

The rectoscope, which is compatible with all laparoscopic equipment and TEM instruments, is connected to a disposable, flexible, single access surgery port (Gloveport, Nelis, Bucheon City, Korea) that has four working channels: three for instruments up to 5 mm in diameter and one for instruments up to 12 mm in diameter such as a laparoscopic camera system. It has also insufflation and venting channels (Fig. 2).

### Experimental study on cadavers

The experimental study was performed on three cadavers (two females and one male respectively 57, 60, and 70 years old). The cadavers were donated to the Human Anatomy Section of the Department of Molecular Medicine of the University of Padua in the context of a Body Donation Program for Anatomical Education and Research [14] and the procedure was carried out in accordance with the European, national, and regional guidelines [15]. This part of the study, which was carried out in 2017, aimed to evaluate the feasibility of the ARAMIS platform and to compare its use with that of two TAMIS single-port surgery devices, SILS Port ( Covidien, Mansifield, MA) and GelPOINT Path Transanal Platform (Applied Medical, CA, USA).

The cadavers were placed in lithotomic position, the rectum was cleansed with multiple hot enemas, and the rectal wall was marked endoscopically with methylene blue 1:10 at 8, 10, 12, and 14 cm from the anal verge.

Endoscopic surgeons with different degrees of experience in laparoscopic colorectal surgery performed running sutures on each cadaver at 4 positions in 4 quadrants (anterior, posterior, right, left) of the rectal wall using ARAMIS, SILS Port, and GelPOINT Path Transanal Platform (with 5.5-cm long proctoscope). The sutures were performed using a 3-0 PDS threat with HR22 needle and closed with silver clips. The surgeons were then asked to rate their perception of the visibility, maneuverability, and the safety of the three platforms on a 3-point scale, with 1 indicating a low rating, 2 a medium one, and 3 a high one (Table 1). The surgeons were also asked to give their opinion on technical aspects such as the instrument’s ease of use and stability.

**Table 1:** Assessment scores: data are presented as mean and standard deviation

Distance	8 cm			10 cm			12 cm			14 cm		
	V	M	S	V	M	S	V	M	S	V	M	S
SILS Port	2.3 ± 0.5	2.6 ± 0.5	1.1 ± 0.3	1.9 ± 0.6	2.4 ± 0.5	1.1 ± 0.2	1.3 ± 0.4	1.3 ± 0.4	1.1 ± 0.2	1.0 ± 0.0	1.1 ± 0.3	1.0 ± 0.0
GelPOINT	2.3 ± 0.7	2.2 ± 0.5	2.9 ± 0.3	2.7 ± 0.4	2.7 ± 0.4	2.4 ± 0.5	2.8 ± 0.4	2.2 ± 0.4	2.3 ± 0.5	2.2 ± 0.7	2.1 ± 0.5	1.7 ± 0.6
ARAMIS	2.1 ± 0.4	2.0 ± 0.4	2.9 ± 0.2	2.8 ± 0.4	2.1 ± 0.5	2.9 ± 0.3	2.8 ± 0.3	2.1 ± 0.2	2.8 ± 0.4	2.5 ± 0.5	1.9 ± 0.6	2.3 ± 0.4

*V* visibility, *M* maneuverability, *S* security

### Pilot clinical study

All the patients at our medical center who underwent TAMIS surgery using the ARAMIS platform between January 2018 and December 2019, after informed consent, were prospectively enrolled in the study. This study was performed in line with the principles of the Declaration of Helsinki. The study protocol was approved by the Ethics Committee of the University Padova (number 4328/AO/17).

The inclusion criteria for transanal minimally invasive surgery were patients with rectal mesenchymal tumors, rectal adenomas, or early rectal cancer that were staged T1, mobile, non-fixed to the rectal wall, well to moderately differentiated (G1–G2), and with no evidence of lymphadenopathy (N0) on pretreatment imaging. All the patients underwent clinical and radiological preoperative staging and colonoscopy with biopsy of the rectal neoplasm. Patients with suspected rectal cancer underwent pelvic MRI, total body CT scan, and blood tests including carcinoembryonic antigen (CEA). All the specimens underwent histopathological examination.

The data collected for our study were the following:

- The patients’ demographic information such as age and sex;
- Description of the neoplasm: its size, histology, distance in centimeters from the anal verge, its position on the rectal wall (anterior, posterior, right, or left);
- Information regarding the procedure: operative times, if the rectal wall was sutured;
- Histopathology: histology, staging, and grading of the lesion and the ease of removing the tumor together with negative margins.

Follow-up examinations, which were scheduled 3, 6, and 12 months after the procedure, included a proctoscopy to identify local recurrence.

### Operative technique

Mechanical bowel preparation with rectal enema is performed before surgery and a single dose of antibiotics (Cefazolin 2 g i.v.) is administered. The procedure is performed under general anesthesia, and the patients are preferably placed in the traditional lithotomy position.

**Fig. 3:**
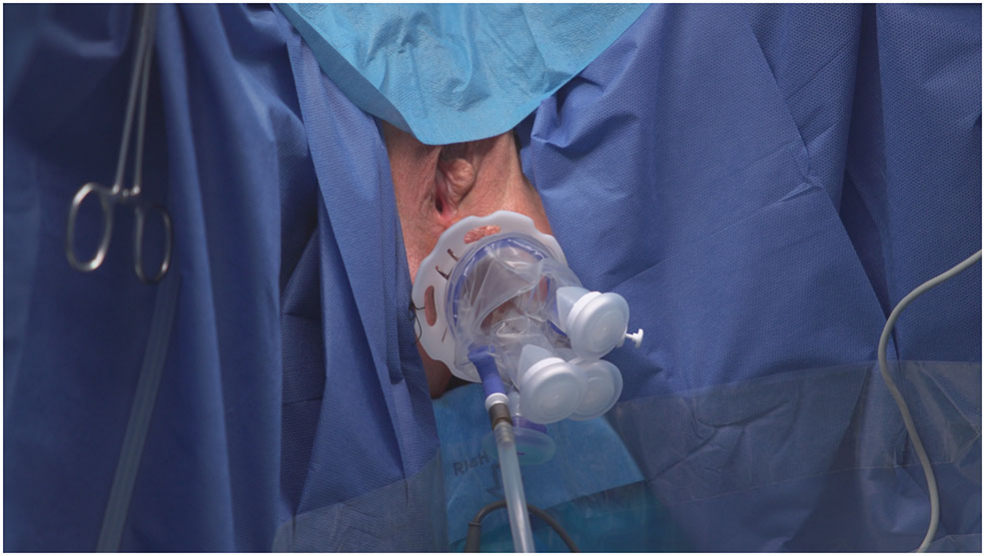
The instrument positioned with fixing ring fixed by stitches to the perianal skin

Perianal skin preparation and sterile draping are carried out following standard protocols. The surgeon must select the appropriate length of the instrument depending on the height of the rectal lesion and position the flute end in the correct direction. The ring is used to regulate the depth, the rectoscope is inserted (one of three possibilities), and the flute opening is turned towards the tumor’s position in the circumference of the rectum. The rectoscope shaft is inserted transanally. When the optimal position of the rectoscope is reached, the fixing ring is fixed by stitches to the perianal skin, and the spin is removed. When the rectoscope is positioned, the port is inserted into the rectoscope and pneumorectum is established by a standard laparoscopic column connected to the port (Thermoflator 264320-20 Karl Storz, Tuttlingen, Germany), with mean pressure of 12–14 mmHg in semicontinuous flow mode (Fig. 3). After a 5-mm camera is placed in one of the channels, the lesion can be excised using laparoscopic instruments including a harmonic or ultrasonic scalpel or TEM equipment inserted into dedicated channels whose diameters measure 5 or 10 mm.

### Statistical analysis

The Freeman-Halton extension of the Fisher’s exact test was carried out for the three rows and three columns contingency table for the cadaver part of the study. *p* values that were < 0.05 were considered statistically significant. Clinical data are presented as means and the ranges are indicated.

## Results

### Cadaver study

Eight endoscopic surgeons with different degrees of laparoscopic experience (range = 1–25 years of experience, a mean of 9 years) used each of the three devices to perform sutures in the four quadrants of the three cadavers at the four distances of 8, 10, 12, and 14 cm. Then, each surgeon performed with each device 16 sutures, for a total of 48 procedures and completed the chart with scores (Table 1). An analysis of their charts revealed that the surgeons rated the visibility provided by the SILS device significantly inferior with respect to that of the GelPOINT Path and ARAMIS for the 10, 12, and 14 cm distances (*p* < 0.05); there were no significant differences for any of the distances between the ARAMIS and GelPOINT Path.

Data analysis showed that the SILS Port provided superior maneuverability for sutures at 8 cm from the anal verge with respect to the GelPOINT Path and ARAMIS (*p* < 0.05); there were no differences with regard to this variable between the GelPOINT Path and ARAMIS. The maneuverability scores were lower for ARAMIS at 10 cm with respect to the SILS Port and GelPOINT Path (*p* < 0.05), whose ratings were similar. They were lower for the SILS Port (*p* < 0.05) at 12 and 14 cm, but the differences between the other two were not statistically different.

The safety scores at 8 from the anal verge were lower for the SILS Port (*p* < 0.05); the other two received similar ratings. The safety scores at 10, 12, and 14 cm were higher for ARAMIS with respect to the other two (*p* < 0.05), but the GelPOINT Path had a higher safety rating with respect to the SILS Port (*p* < 0.05). The different degrees of laparoscopic experience had no significant impact on the reported scores.

### Qualitative aspects

Positioning the ARAMIS resulted easy and rapid. Although the rectoscope provides stability to the pneumorectum and ensures protection during surgical procedures, it limits maneuverability. Nevertheless, as the port is flexible, even instruments with a curved tip can be inserted. The rectoscope also guarantees a good vision of the operating field since the depth of insertion and the angle of vision of the distal opening with 45° of inclination can be adjusted. Proximal rectal neoplasms can be easily reached as the rectoscope is able to straighten the rectal walls and its anatomical curves.

Insertion of ARAMIS is facilitated by a spindle within the rectoscope. Its capacity to establish and maintain pneumorectum can be considered optimal since the rectoscope adheres to the rectal walls. The rectoscope, which is connected to an external fixing ring secured to the perineum by 4 stitches, provides stability during surgery. After removal of the port, it is easy to extract the tumor, needle assembly to the needle holder or perfecting the suture transanally, if required.

### Pilot clinical study

The instrument could not be positioned in one of the candidates for ARAMIS because of an anal stricture linked to previous anal surgery; we resorted to an endoscopic procedure in that case. In the other 14 (93%) patients, the procedure was completed without conversion to other approaches despite the fact that there were 5 tumors located 11 cm from the anal verge. The procedure lasted a mean of 59 min (range 25–110). Patient and tumor data are summarized in Table 2.

**Table 2:** Characteristics of the patients who underwent ARAMIS and details regarding the procedure and the lesion

	Age	Sex	Distance (cm)	Position	Dimension (cm)	Suture	Duration (min)	Histology	Complete removal
1	45	F	9	Post-R	2	No	90	LGD	Yes
2	64	M	9	Ant-L	2	Yes	67	pT1G2	Yes
3	71	M	9	Left	3	Yes	80	LGD	Yes
4	74	F	13	Ant	5	No	60	LGD	Yes
5	73	M	8	Ant-L	3	Yes	110	LGD	Yes
6	67	F	15	Post-R	3	No	105	Anastomotic cancer recurrence	No
7	Not possible rectoscope position								
8	66	F	10	Post-R	3	Yes	50	LGD	Yes
9	74	F	8	Left	3	No	45	LGD	Yes
10	62	F	13	Ant	4	No	25	HGD	Yes
11	69	M	15	Post-L	3	No	30	pT1G1	Yes
12	60	F	15	Left	2	No	35	LGD	Yes
13	70	F	8	Ant	4	Yes	60	LGD	Yes
14	80	M	8	Ant-L	3	Yes	35	LGD	Yes
15	68	F	8	Post	2	Yes	30	Lipoma	Yes
Mean range	67 years (45–80)		11 cm (8–15)		3 cm (2–5)		59 min (25–110)		

*F* female, *HGD* high grade dysplasia, *L* left, *LGD* low grade dysplasia, *M* male, *Post* posterior, *R* right

There were no intraoperative or postoperative complications. The lesions extracted were 3 invasive carcinomas, 2 high grade dysplasia (HGD) polyps, 8 low grade dysplasia (LGD) polyps, and 1 lipoma. In all but one case, the patients underwent the procedure in gynecologic position including 5 patients whose tumors were positioned anteriorly. In only one case presenting an anterior tumor, the procedure was performed in a prone position.

The margins were sutured in 7 patients: a continuous PDS suture fixed with a silver clip was carried out in 4 and single stitches under direct vision through the ARAMIS rectoscope were sewn after the port was removed in three. Rectal wall was closed after full-thickness excision in every patient.

The lesion was entirely removed in 13/14 patients (93%). It was removed incompletely (positive margins) in one patient who presented cancer recurrence at an anastomosis site located 15 cm from the anal verge. It proved difficult to remove the tumor completely in that case because it had grown through the anastomotic clips. The patient later underwent total mesorectal excision for rectal cancer.

All the patients who underwent the procedure were monitored for a 12-month period. None of the patients reported incontinence or urgency at the follow-up examinations. There was one (8%) recurrence out of the 13 cases of complete removal; a small (3–4 mm) LGD adenoma was found and removed through flexible proctosigmoidoscopy 3 months after a 4-cm large polyp had been removed with ARAMIS.

## Discussion

A number of transanal surgical platforms have been designed and developed in the effort to avoid invasive rectal surgery and the risk of an intestinal stoma or of functional disabilities. While local transanal excision is associated to high rates of oncologic failures [16–18], recent studies have demonstrated that TEM and TAMIS transanal endoscopic microsurgery techniques allow access to rectal lesions and ensure high-quality full-thickness excisions whose oncologic outcomes are comparable with anterior rectal resection or the Miles procedure [19–22]. According to a large meta-analysis by Clancy et al.,^23^ TEM is better than transanal local excision as far as oncological outcomes, such as margin clearance, specimen fragmentation, and local recurrence, are concerned.

Minimally invasive surgery based on endoscopic platforms is now developing to ensure better visualization and precision during local excision for benign neoplasm or early rectal cancer, and also better oncological results than simple transanal excision [23]. In addition, transanal endoscopic platforms are able to reach more deeply located lesions with respect to classical transanal operations [24].

When Melin et al. [25] retrospectively reviewed TEM and TAMIS, they reported that the two procedures had equivalent indications and outcomes although the specimens were larger and mesorectal lymphonodes were more frequent in the TAMIS group. While TEM provides limited visibility and enables operations on only a single quadrant of the rectal wall, TAMIS ensures better maneuverability and 360° visualization of the rectal wall without repositioning the patient or instruments during surgery thus facilitating local radical en-bloc resection of the mesorectum and transanal total mesorectal excision (TaTME) [26]. One of the limits of the TAMIS platform is that it is conceived to cross the anus which makes it more difficult to excise upper rectal lesions since there is no rectoscope to protect the rectum.

ARAMIS, a novel disposable instrument composed of a rectoscope and a laparoscopic interface, is presented here. According to our study’s results, the platform can reach and excise rectal tumors located as far as 15 cm from the anal verge. In addition, the rectoscope protects the rectal wall from being damaged during the procedure, and it guarantees a good visibility of the field of view since it is possible to adjust the depth of insertion and the angle of vision provided by the distal opening with a 45° inclination angle. Proximal rectal neoplasms are easily reached by the rectoscope which is also able to straighten the rectal walls and its anatomical curves. This straightening, combined with the possibility to turn the wider flute opening towards the tumor position, can selectively improve visibility in the surgery site. The presence of a rigid rectoscope also reduces the phenomenon of billowing pneumorectum even in absence of a continuous insufflation.

In addition, the rectoscope can be used without the laparoscopic interface for some procedures, including suturing.

ARAMIS shares a few features with some TEM/transanal endoscopic operation (TEO) (Storz, Tuttlingen, Germany) and TAMIS instruments. It is similar to TEM because (1) it is composed of a rigid rectoscope (the diameter of the ARAMIS rectoscope—35.5 mm—is smaller than the TEM’s—40 mm) which protects the rectal wall during surgery and guarantees a safe resection of proximal lesions; (2) the rectoscope ensures stability; and (3) it is compatible with all TEM instruments. ARAMIS is similar to TAMIS instruments because (1) it can be used with standard laparoscopic instruments. (2) There is no need to secure the rectoscope to the operating table because a secure ring is stitched to the perineum. (3) The angled distal opening of the rectoscope allows a wide view of the operating field. (4) The camera can be operated freely. (5) The rectoscope can be rotated and inserted more deeply if necessary which makes it possible to reach all rectal locations without moving the patient. It is thus possible to operate patients in a gynecologic position for anteriorly located tumors. (6) The instrument is disposable. A limit observed when compared with the other TAMIS devices was less instrumental maneuverability at 10 cm distance due to the rigid rectoscope. This was not reported for longer distances.

The results of the current study demonstrate that ARAMIS is a safe, effective platform for transanal surgery to remove tumors of the upper third of the rectum. As it is easy to assemble, the procedure is rapid, lasting in mean of 59 min, a time which is consistent with the durations of TAMIS procedures (55–86 min) reported in the literature. Our results with regard to its efficacy are consistent with studies evaluating the TAMIS technique. Atallah et al.’s [6] first study reporting on 6 patients who underwent a TAMIS procedure described no complications or need to convert to anterior resection or to the Miles operation. A second study examining 50 patients [20] reported 6% of positive margins, 6% of early complications, no late complications, and 2 recurrences of neoplasms at the follow-up examination carried out 20 months later.

In their systematic review of 390 TAMIS procedures, Martin-Perez et al. [11] found a 2.3%, conversion rate, a 4.36 % rate of positive margins, and a 4.1% rate of specimen fragmentation. There was a 7.4% complication rate, and the median tumor size was 3 cm. The mean distance of the rectal lesions from the anal verge was 7.6 cm; in our patients, it was 11 cm.

When Keller et al. [27] reviewed a prospective database of patients who underwent TAMIS in a single center using two platforms (GelPOINT Path Transanal Access, Applied Medical and SILS Port, Covidien), they found a mean operative time of 69 min and concluded that the procedure minimizes morbidity and enables more patients to benefit from the minimally invasive approach.

In conclusion, our study’s results demonstrate that ARAMIS is a safe, effective platform for transanal minimally invasive surgery whose main advantage with respect to others is that it has a built-in rectoscope. As the study examined only a limited number of cases mainly involving benign lesions, further clinical studies are warranted to confirm these encouraging results regarding the safety, maneuverability, efficacy, and operator-friendliness of the procedure. A comparison with other devices in the clinical setting by means of evaluation scores is also desirable.

## Data Availability

Data of experimental and clinical study are available.
